# Effect of bradykinin on nitric oxide production, urea synthesis and viability of rat hepatocyte cultures

**DOI:** 10.1186/1472-6793-5-2

**Published:** 2005-01-25

**Authors:** Settimio Sesti, Guglielmo Martino, Sergio Mazzulla, Rosa Chimenti

**Affiliations:** 1 Department of Cell Biology, University of Calabria, Italy

## Abstract

**Background:**

It is well known that cytotoxic factors, such as lipopolysaccharides, derange nitrogen metabolism in hepatocytes and nitric oxide (NO) is involved among the other factors regulating this metabolic pathway. Hepatocytes have been shown to express large levels of NO following exposure to endotoxins, such as bacterial lipopolysaccharide and/or cytokines, such as tumour necrosis factor-α (TNFα), interleukin-1. The control role of arginine in both urea and NO biosynthesis is well known, when NO is synthesized from arginine, by the NOS reaction, citrulline is produced. Thus, the urea cycle is bypassed by the NOS reaction. Many authors demonstrated in other cellular types, like cardiomyocytes, that bradykinin caused the increase in reactive oxygen species (ROS) generation. The simultaneous increase of NO and ROS levels could cause peroxynitrite synthesis, inducing damage and reducing cell viability. The aim of this research is to study the effect of bradykinin, a proinflammatory mediator, on cell viability and on urea production in cultures of rat hepatocytes.

**Results:**

Hepatocytes were treated with bradykinin, that stimulates nitric oxide synthase (NOS). NO release was determined using 4,5 diaminofluorescein diacetate (DAF-2DA), as fluorescent indicator of NO. Addition of the NOS inhibitor, N^g^-nitro-L-arginine methyl ester (L-NAME), to the culture medium inhibited the increase of NO production. Exposure of hepatocytes to bradykinin 0,1 mM for 2 hours resulted in a significant decrease of urea synthesis. Cell viability, instead, showed a significant decrease 24 hours after the end of bradykinin treatment as determined by 3-(4,5-dimethyl-2-thiazolyl)-2,5diphenyl-2H-tetrazolium (MTT) assay. L-NAME addition recovered urea production and cell viability at control values.

**Conclusion:**

The findings suggest that the cell toxicity, after bradykinin treatment, effectively depends upon exposure to increased NO levels and the effects are prevented by L-NAME. The results show also that the increased NO synthesis induces a reduced urea production, that is another index of cell damage.

## Background

It is well known that cytotoxic factors, such as lipopolysaccharides, derange nitrogen metabolism in hepatocytes and nitric oxide (NO) is involved among the other factors regulating this metabolic pathway [1]. NO is a free radical that is involved in many cellular events. In the biological systems NO has an halflife long lasting few seconds. It is an oxidation intermediate, therefore is both an oxidant and a reducing agent of metabolic products. Its biosynthesis is mainly performed by converting L-arginine to L-citrulline. L-arginine analogues, such as N^g^-nitro-L-arginine methyl ester (L-NAME), act as false substrates and are selective inhibitors of NO synthesis. NO synthase (NOS) is either a constitutive or inducible enzyme. The endothelial isoform (e-NOS) and the neuronal isoform (n-NOS) are constitutive. The inducible form of the enzyme (i-NOS), has the main property to be not regulated by intracellular calcium concentration and Ca^2+^-calmodulin complex, unlike the constitutive form [2]. It is known that iNOS is expressed by many cell types including macrophages, smooth muscle cells and hepatocytes [3]. Hepatocytes have been shown to express large levels of NO following exposure to endotoxins, such as bacterial lipopolysaccharide and/or cytokines, such as tumour necrosis factor-α (TNFα), interleukin-1 [4,5]. NO may posses both cytoprotective and cytotoxic properties, depending on the amount and the isoform of NOS by which it is produced [6]. NO generally mediates beneficial responses, but becomes deleterious when coexistence with enhanced superoxide formation leads to the synthesis of peroxynitrite, a potent oxidant and nitrating agent [7]. According to this hypothesis, authors studied the effect of bradykinin, a proinflammatory mediator kinin, on cell viability and on urea production in cultures of rat hepatocytes. Kinins exert numerous physiological and pathological actions; they partecipate in vascular and cellular events that accompany the inflammatory processes. In pathological states, kinins are thought to be implicated in inflammatory diseases and in haemorrhagic and endotoxic shock [8]. To demonstrate the decrease of cell viability and urea production by bradykinin, the authors studied its effects on NO production. The measurements of NO release from hepatocytes were investigated by using a NO-specific fluorescence indicator, 4,5 diaminofluorescein diacetate (DAF-2DA) [9].

## Results

### Effect of bradykinin treatment on NO production

**Figure 1:**
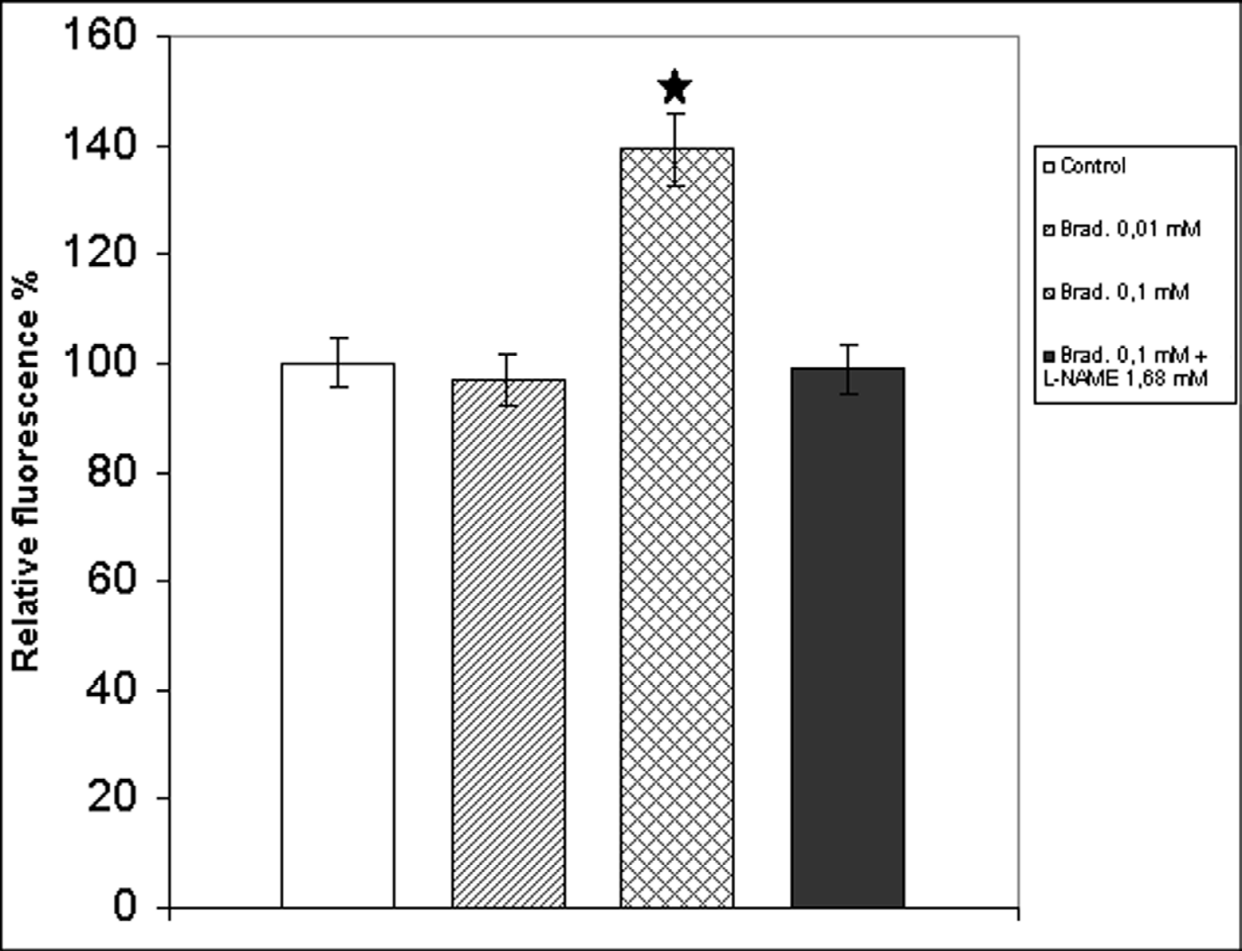
Determination of NO release after treatment of hepatocytes with bradykinin. Fluorescence intensity was measured after 2 h incubation: with 10 μM DAF-2DA in basal conditions (white column, reference), in presence of 0.01 mM bradykinin (hatched column), in presence of 0.1 mM bradykinin (crosshatched column) and in presence of 0.1 mM bradykinin with 1.68 mM L-NAME (black column). The excitation wavelength was 495 nm and the emission wavelength was 515 nm. Values, expressed as a percentage of control values, are the means ± S.E.M. (bars) of four independent experiments. 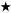 p < 0,05 compared with control

The amounts of released NO were measured using DAF-2DA, that specifically reacts with the oxidized form of NO, producing the fluorescent triazolofluorescein [9]. NO determination was performed after 2 hours of incubation in the presence of bradykinin (0.01 mM and 0.1 mM). As shown in figure 1 the treatment with 0.01 mM bradykinin did not produce NO increase compared to control, but 0.1 mM bradykinin increased significantly the NO release. In contrast no appreciable NO release was observed during the same period in hepatocytes cultured with 0.1 mM bradykinin and 1.68 mM L-NAME.

### Effect of bradykinin treatment on urea production

**Figure 2:**
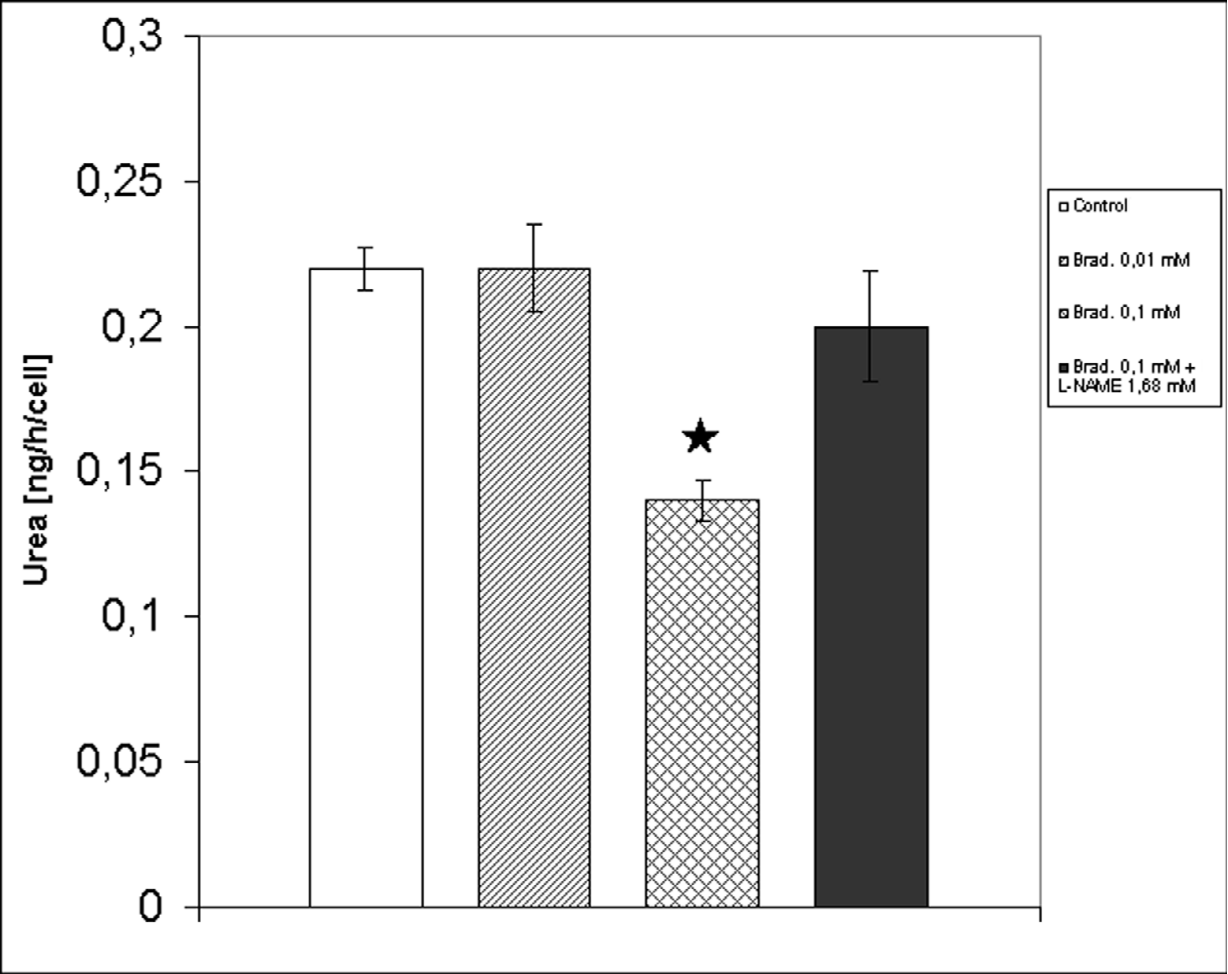
Determination of urea production after treatment of hepatocytes with bradykinin. Urea production was spectrophotometrically determined at 600 nm after 2 h incubation with 1 mM NH_4_Cl in basal conditions (white column), in presence of 0.01 mM bradykinin (hatched column), in presence of 0.1 mM bradykinin (crosshatched column) and in presence of 0.1 mM bradykinin with 1.68 mM L-NAME (black column). Values, expressed as ng urea per cell per hour, are the means ± S.E.M. (bars) of four independent experiments. 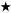 p < 0,05 compared with control

To evaluate urea synthesis after bradykinin treatment, the hepatocytes were treated with 1 mM NH_4_Cl for 2 h. Figure 2 shows that only the treatment with 0.1 mM bradykinin significantly decreased urea production and that the treatment with 0.1 mM bradykinin and 1.68 mM L-NAME did not produce a significant urea level decrease in comparison to control.

### Effect of bradykinin treatment on cell viability

**Figure 3:**
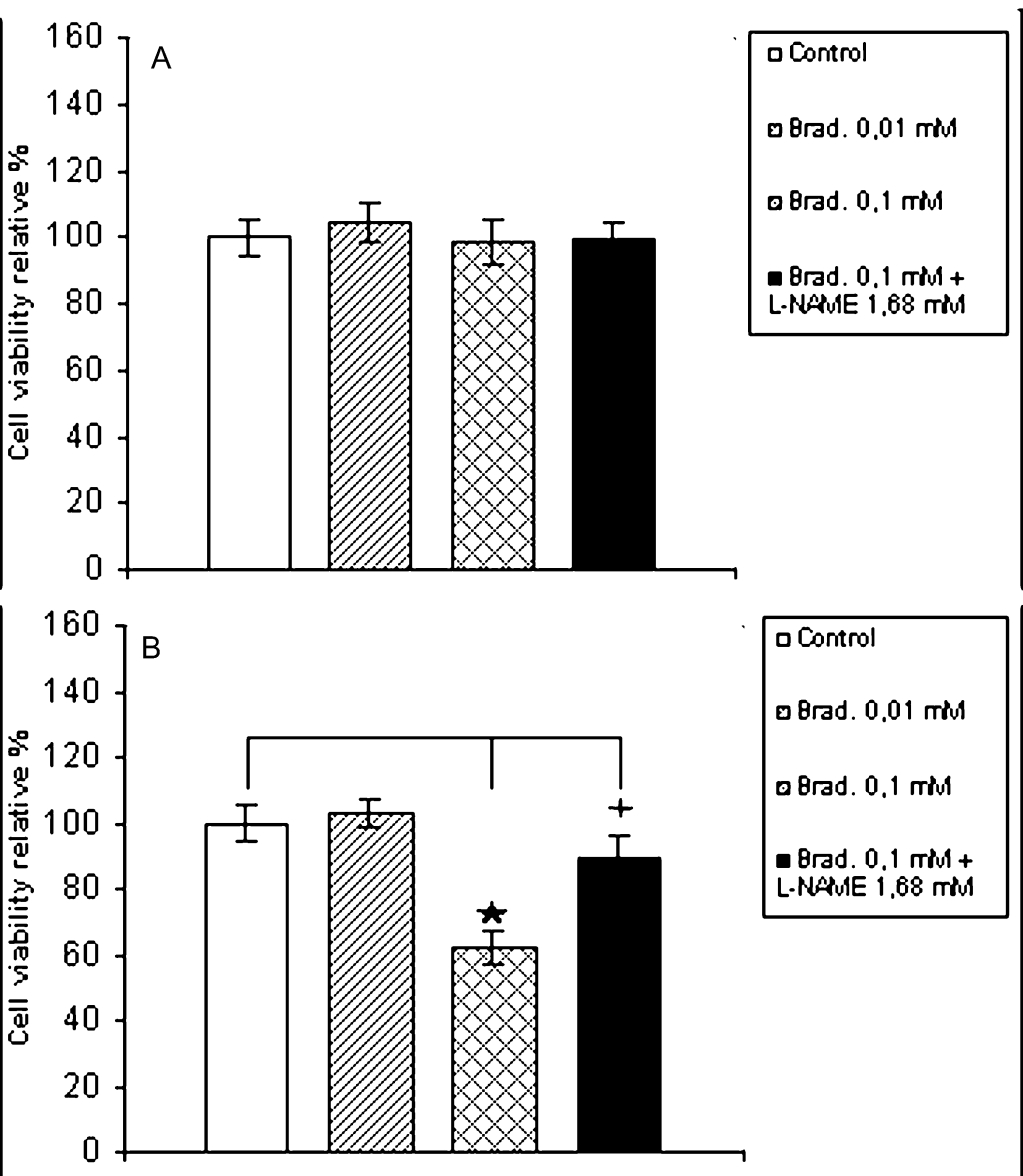
Determination of cell viability in hepatocytes treated with bradykinin. The cell viability was spectrophotometrically determined at 570 nm by MTT assay in hepatocytes incubated in basal conditions (white column), in presence of 0.01 mM bradykinin (hatched column), in presence of 0.1 mM bradykinin (crosshatched column) and in presence of 0.1 mM bradykinin with 1.68 mM L-NAME (black column) for 2 h period. (A) Cell viability determined immediately after. (B) Cell viability determined after an additional 24 h incubation period in incomplete medium Results are expressed as a percentage of control. Values are the means ± S.E.M. (bars) of four independent experiments. 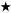 p < 0,05 compared with control. 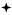 p < 0,05 compared with control and 0.1 mM bradykinin treated cells

To determine the effects of bradykinin on cell viability, the hepatocytes were exposed to bradykinin (either 0.01 mM or 0.1 mM) for an incubation time of 2 hours. In one experimental series, the cell viability was determined by MTT test after 2 hours of incubation. In a second one, culture medium containing bradykinin was removed and replaced with the same fresh medium at 2 hours after the addition of bradykinin, and then cell viability was measured 24 hours after the end of bradykinin treatment. The MTT test after 2 hours of incubation does not indicate any significant viability difference in treated hepatocyte cultures in comparison to control (figure 3A). By MTT test after 24 h (figure 3B), a significant lowering of viability is observed in bradykinin 0.1 mM treated hepatocytes in comparison to control. The decrease was significantly reduced by the simultaneous treatment with L-NAME 1.68 mM even if always significantly lower than in control. Cell viability was validated by Trypan blue exclusion test (Table 1).

## Discussion

The role of NO as mediator of hepatic injury after endotoxic shock remains controversial [16]. Increased NO production in response to cytokines has been demonstrated in cultured hepatocytes [17]. Laskin et al. [18] demonstrated that the induction of acute endotoxemia, caused an increase in NO production in the liver. This was associated with expression of inducible nitric oxide synthase (iNOS) messenger m-RNA in hepatocytes. Also our data showed an increase of NO production after 2 hours treatment of culture with 0.1 mM bradykinin in an arginine supplemented medium, as substrate for the synthesis of NO. The simultaneous treatment with L-NAME, a known inhibitor of NOS, blocked the increase of NO production. In this work we analyzed the urea synthesis after bradykinin treatment. Urea synthesis was decreased after 2 hours treatment with bradykinin 0.1 mM and the simultaneous treatment with L-NAME leaves urea biosynthesis unaltered. These data can be attributed to the control role of arginine in both urea and NO biosynthesis. When NO is synthesized from arginine, by the NOS reaction, cytrulline, an intermediate of urea cycle, is produced. Thus, the urea cycle is bypassed by the NOS reaction [1]. Whether NO exerts cytotoxic or cytoprotective action remains unclear [6]. We also found a significant decrease of viability, at long term, in hepatocytes subjected to bradykinin treatment. The simultaneous treatment of hepatocytes with L-NAME improves cell viability even if control levels are not restored. The data show that the increased NO production plays a role in liver damage induction, that follows the proinflammatory mediator treatment. The hepatocellular injury attributed to NO may be due either to its direct cytotoxicity or its reaction with superoxide to produce the toxic nitrogen metabolite peroxynitrite [19]. Oldenburg et al. [20], demonstrated in other cell types, like cardiomyocytes, that bradykinin caused the increase in reactive oxygen species (ROS) generation. At last, our results show that the increased NO synthesis induces a reduced urea production, that is an index of cell damage. The simultaneous treatment of liver cell cultures with L-NAME decreases NO levels and sustains overall biosynthesis activities and cell viability.

## Conclusions

In summary, we conclude that 0.1 mM bradykinin treatment induces an increase of NO levels and reduction of urea synthesis in the hepatocytes. This increased NO production mediates, after 24 hours, cell toxicity as shown by MTT test. In contrast, the administration of the NOS inhibitor L-NAME protects against cell damage and increases urea levels, suggesting that NO plays a key role in the bradykinin-induced liver damage.

## Methods

### Materials

Unless otherwise specified, all chemicals were obtained from Sigma (St. Louis, MO, USA).

### Isolation and culture of rat hepatocytes

Hepatocytes were isolated from male rats, Wistar strain, (180 to 200 gbw), by a modification of the method of Seglen [10]. All procedures on the animals were performed according to the CEE directive n. 86/609 on animal experimentation. Rats were anesthetized with diethylether, the pre-perfusion of the liver in situ was performed at a rate of 20–30 ml/min with Ca^2+^-free Hanks balanced salt solution. The liver was then excised and the digestion was carried out by adding 0.05% (w/v) collagenase (type IV) in Hanks balanced salt solution supplemented with CaCl_2_·H_2_O (0.0186 g/L) at a flux rate of 40 ml/min. At this point liver was transferred to a square plate containing 100 ml of RPMI 1640 medium supplemented with 200 mM L-glutamine, 20 ml/L essential amino acid solution and 10 ml/L non-essential amino acid solution, 1% antibiotic antimycotic stabilized solution and 100 μM L-arginine (incomplete medium). The cells were dispersed by gentle distruption with a stainless steel comb. After filtration through 200 μm Nytal mesh, parenchymal cells (hepatocytes) were separated from nonparenchymal cells (endothelial cells, Kupffer cells and stellate cells) by centrifugation at 50 g in Eppendorf Centrifuge 5810R at 4°C for 2 minutes and then washed twice in washing buffer [11]. Then the cells were resuspended in the same medium and filtered through 63 μm Nytal mesh. The viability of the cells was more than 80%, as estimated by trypan blue dye exclusion test [12]. After cell counting the cells were diluited at a concentration of 5 × 10^5 ^cells/ml with incomplete medium supplemented with 2% fetal calf serum, 0.1 U/ml insulin and 10^-6 ^M dexamethasone (complete medium). The hepatocytes were then plated in 24 well-plates coated with rat tail collagen at the final cell density of 2.5 × 10^5 ^cells per well and incubated at 37°C in an humidified atmosphere of 5% CO_2 _and 95% air. After 6 hours incubation, the medium was changed and replaced with incomplete medium to remove dead cells.

To verify the isolation method efficiency, the acid fosfatase activity per mg of proteins was evaluated. According to literature data, the specific activity of acid fosfatase in nonparenchimal cells is 1,7 folds the same activity in parenchimal cells [13].

### Treatment

After 24 hours of culture the hepatocytes were exposed either to bradykinin (0.01 mM and 0.1 mM) or bradykinin 0.1 mM supplemented with L-NAME 1.68 mM [14].

### Determination of NO from hepatocytes

DAF-2DA (Alexis Biochemicals, Lausen, Switzerland) was dissolved in DMSO (1 mg/0.45 ml) and diluted to 10 μM in phosphate buffer (0.1 M, pH 7.4). Then the cells were either incubated in phosphate buffer containing 10 μM DAF-2DA, bradykinin (0.01 mM and 0.1 mM) and bradykinin 0.1 mM supplemented with L-NAME 1.68 mM. After 2 hours of incubation in this reaction mixture, the fluorescence from the reaction of DAF-2DA with NO released from hepatocytes was measured with Perkin-Elmer MPF-44B Spectrofluorimeter calibrated for excitation at 495 nm and emission at 515 nm. Results were expressed as a percentage of the fluorescence of the samples in comparison to control.

### Determination of urea synthesis

To determine the effects of bradykinin on urea production, cells were treated either with bradykinin (0.01 mM and 0.1 mM) and cotreated with bradykinin 0.1 mM and L-NAME 1.68 mM. At the same media 1 mM NH_4_Cl was added. After 2 hours urea levels in the media were measured by spectrophotometric method using Urea Color 2 Kit (Sclavo Diagnostics, Siena, Italia) measuring absorbance at 600 nm and blank sample with the same NH_4_Cl final concentration was used. Urea synthesis was calculated as ng urea per cell per hour.

### Determination of cell viability

Cell viability was determined by MTT test method [15] and confirmed by Trypan blue exclusion test [12]. MTT (5 mg/ml) was dissolved in RPMI-1640 without phenol red. The solution is filtered through a 0.2 μm filter and stored at 2–8°C for frequent use. To determine the effects of bradykinin on cell viability, cells were either treated with bradykinin (0.01 mM and 0.1 mM) and cotreated with bradykinin 0.1 mM and L-NAME 1.68 mM for a 2 h period. After that cells were used either immediately or after an additional 24 h incubation period in incomplete medium. For the determination of cell viability, the medium has been discarded and MTT solution was added and incubated for 3 hours. At the end of the incubation period the MTT solution was removed and the cells and dye cristals were dissolved by adding dimethylsulfoxide (DMSO). Absorbance was measured at 570 nm in a Shimadzu UV-2100 Spectrophotometer and the results were expressed as a percentage of the absorbance of the samples in comparison to control.

### Statistical analysis

At least four independent determinations of each parameter were compared to control using Student's T-test. Differences were considered significant when p < 0.05 was obtained.

## Authors' contributions

SS: Fluorimetric analysis and overall statistical analysis of data

MG: Director of research

MS: Spectrophotometric analysis

CR: Primary hepatocyte cultures and characterization

All authors read and approved the final manuscript

## Figures and Tables

**Table 1:** Effect of bradykinin treatment on cell viability

	Treatment: 2 h+Brad (Viability %)	Treatment: 2 h+Brad + 24 h-Brad (Viability %)
Control	100 ± 10	100 ± 6
0.01 mM Bradykinin	98 ± 12	104 ± 10
0.1 mM Bradykinin	104 ± 8	50 ± 11*
0.1 mM Bradykinin + 1.68 mM L-NAME	102 ± 10	79 ± 7**

Hepatocytes were isolated and cultured in presence and in absence of bradykinin (either 0.01 mM or 0.1 mM) and cotreated with bradykinin 0.1 mM and L-NAME 1.68 mM for a 2 h period. Cell viability was determined by Trypan blue exclusion test either immediately after 2 h or after additional 24 h incubation period in incomplete medium. Results are expressed as a percentage of control. Values are expressed as mean ± S.E.M., n = 4.

*Statistically significant differences (p < 0,05) from control levels as determined by Student's t-test.

** Statistically significant differences (p < 0,05) from control levels and from 0.1 mM bradykinin treated cells as determined by Student's t-test.
